# Multiscale, Converging Defects of Macro-Porosity, Microstructure and Matrix Mineralization Impact Long Bone Fragility in NF1

**DOI:** 10.1371/journal.pone.0086115

**Published:** 2014-01-21

**Authors:** Jirko Kühnisch, Jong Seto, Claudia Lange, Susanne Schrof, Sabine Stumpp, Karolina Kobus, Julia Grohmann, Nadine Kossler, Peter Varga, Monika Osswald, Denise Emmerich, Sigrid Tinschert, Falk Thielemann, Georg Duda, Wenke Seifert, Thaqif el Khassawna, David A. Stevenson, Florent Elefteriou, Uwe Kornak, Kay Raum, Peter Fratzl, Stefan Mundlos, Mateusz Kolanczyk

**Affiliations:** 1 Institute for Medical Genetics and Human Genetics, Charité - Universitätsmedizin Berlin, Berlin, Germany; 2 FG Development & Disease, Max Planck Institute for Molecular Genetics, Berlin, Germany; 3 Department of Biomaterials, Max Planck Institute for Colloids and Interfaces, Potsdam, Germany; 4 Department of Chemistry, Universität Konstanz, Konstanz, Germany; 5 Institut für Physiologische Chemie, MTZ, Medizinische Fakultät Carl Gustav Carus, Technische Universität Dresden, Dresden, Germany; 6 Julius Wolff Institute & Brandenburg School of Regenerative Therapies, Charité - Universitätsmedizin Berlin, Berlin, Germany; 7 Division für Humangenetik, Medizinische Universität Innsbruck, Innsbruck, Austria; 8 Institut für Klinische Genetik, Medizinische Fakultät Carl Gustav Carus, Technische Universität Dresden, Dresden, Germany; 9 Klinik für Orthopädie, Medizinische Fakultät Carl Gustav Carus, Technische Universität Dresden, Dresden, Germany; 10 Berlin-Brandenburg Center for Regenerative Therapies, Berlin, Germany; 11 Institute for Vegetative Anatomy, Charité - Universitätsmedizin Berlin, Berlin, Germany; 12 Laboratory of Experimental Trauma Surgery Giessen, Justus-Liebig University Giessen, Giessen, Germany; 13 University of Utah, Department of Pediatrics, Division of Medical Genetics, Salt Lake City, Utah, United States of America; 14 Department of Medicine, Pharmacology and Cancer Biology, Center for Bone Biology, Vanderbilt University - Medical Center, Nashville, Tennessee, United States of America; INSERM U606 - university Paris 7, France

## Abstract

Bone fragility due to osteopenia, osteoporosis or debilitating focal skeletal dysplasias is a frequent observation in the Mendelian disease Neurofibromatosis type 1 (NF1). To determine the mechanisms underlying bone fragility in NF1 we analyzed two conditional mouse models, Nf1Prx1 (limb knock-out) and Nf1Col1 (osteoblast specific knock-out), as well as cortical bone samples from individuals with NF1. We examined mouse bone tissue with micro-computed tomography, qualitative and quantitative histology, mechanical tensile analysis, small-angle X-ray scattering (SAXS), energy dispersive X-ray spectroscopy (EDX), and scanning acoustic microscopy (SAM). In cortical bone of Nf1Prx1 mice we detected ectopic blood vessels that were associated with diaphyseal mineralization defects. Defective mineral binding in the proximity of blood vessels was most likely due to impaired bone collagen formation, as these areas were completely devoid of acidic matrix proteins and contained thin collagen fibers. Additionally, we found significantly reduced mechanical strength of the bone material, which was partially caused by increased osteocyte volume. Consistent with these observations, bone samples from individuals with NF1 and tibial dysplasia showed increased osteocyte lacuna volume. Reduced mechanical properties were associated with diminished matrix stiffness, as determined by SAM. In line with these observations, bone tissue from individuals with NF1 and tibial dysplasia showed heterogeneous mineralization and reduced collagen fiber thickness and packaging. Collectively, the data indicate that bone fragility in NF1 tibial dysplasia is partly due to an increased osteocyte-related micro-porosity, hypomineralization, a generalized defect of organic matrix formation, exacerbated in the regions of tensional and bending force integration, and finally persistence of ectopic blood vessels associated with localized macro-porotic bone lesions.

## Introduction

Neurofibromatosis type 1 (NF1) is an autosomal dominant disease caused by loss of function mutations in the *NF1* gene, which encodes the Ras-GAP protein neurofibromin. NF1 is frequently associated with skeletal manifestations such as osteopenia, osteoporosis or debilitating focal skeletal dysplasias [1], [2], [3], [4], [5]. *NF1* ablation results in activation of canonical mitogen-activated protein kinase (MAPK) signaling. Activation of MAPK signaling modifies PI3K-mTOR, c-Jun N-terminal kinase (JNK), or JAK-STAT3 signaling, dependent on the cellular context [6], [7]. Loss of *NF1* also impacts cAMP-PKA [8], Rho-ROCK-LIMK2-Cofilin [9], and Rac1-Pak1-LIMK1-Cofilin [10] pathways. We have previously shown that conditional inactivation of *Nf1* in mesenchymal progenitor cells of developing limbs (Nf1Prx1) results in diminished long bone growth, tibial bowing, hip joint fusion, and muscle dystrophy [11], [12]. Skeletal dysplasia in Nf1Prx1 mice is primarily caused by decreased proliferation and premature hypertrophy of the growth plate chondrocytes and secondly by increased osteoblast proliferation and impaired differentiation [11]. The muscle abnormalities and weakness we observed appear to aggravate the tibial dysplasia phenotype in Nf1Prx1 mice [12]. A much milder bone phenotype, characterized by hyperosteoidosis and high bone turnover, is observed in Nf1Col1 mice. Ablation of *Nf1* in Nf1Col1 mice occurs at the pre-osteoblast stage and is restricted to bone forming cells [13]. Our current view of the NF1 bone pathology, based on the analysis of growth plate chondrocyte, osteoblast and osteoclast dysfunction, is that increased bone turnover and an imbalance between extracellular matrix (ECM) synthesis and mineralization cause the NF1 skeletal manifestations [11], [13], [14], [15]. However, our understanding of the mechanisms underlying long bone fragility in NF1 remains incomplete.

The ultimate strength of individual skeletal elements is determined by several factors, including organ morphology, microscopic tissue structure and molecular organic/inorganic matrix composition [16], [17], [18]. Long bones typically develop through the process of endochondral ossification, where a cartilaginous template is progressively replaced by trabecular and cortical bone [19], [20]. During embryogenesis, bone formation is preceded by blood vessel invasion of the cartilaginous template and consecutive infiltration by mesenchymal and haematopoietic progenitor cells [21], [22]. Subsequently, soft tissue templates are entirely replaced with mineralized matrix and most blood vessels are removed [19]. In adult human bones the Haversian system, a network of ∼50–100 µm diameter wide channels, facilitates blood supply. These structures are continuously remodeled and account for an estimated macro-porosity of about 5% in the diaphyseal human bone cortex volume [20].

Osteoclast- and osteoblast-driven remodeling of the lacuno-canallicular system is coordinated by osteocytes, the predominant cell type in adult cortical bone (>90%), conferring bone its capability to sense and to respond to mechanical stimuli. Thus, bone strength is critically controlled by osteocytes, which determine micro-porosity, degree of bone matrix formation and quantity as well as properties of deposited mineral [16], [17], [23], [24], [25]. Depending on species and age, the volume fraction of osteocyte lacunae in the mammalian cortical bone can vary between 1 and 4% [24], [26]. Results of mechanical testing suggest a strong correlation between the osteocyte fraction volume and the elastic modulus [24], [26]. However, larger defects originating from bone mineral lesions, or abundant blood vessels, exert a presumably higher impact on the elastic modulus and ultimate strength. On the micro-structural level, bone tissue is a composite of a collagenous framework, integrated small proteins, and the mineral phase formed by carbonated hydroxyapatite (HA) [18], [20], [23]. The HA mineral phase provides strength and stiffness, while the collagen framework mainly confers toughness [27]. Defects in any one of these components can cause low bone mass and/or diminished bone quality, resulting in bone fragility and increased fracture risk.

Here we apply a combination of biophysical, histological and molecular techniques to give insights into bone structure, micromechanical parameters and material properties. We also assess the underlying cellular changes of bone fragility in the context of *Nf1* deficiency in the mesenchymal lineage. Our analysis sheds light on the previously unrecognized role of osteocytes and blood vessels in the etiology of bone micro-structure and focal cortical bone mineralization defects in Nf1Prx1 mice. These findings suggest that interactions of *Nf1* deficient osteoblasts, osteocytes and blood vessels are important to the development of fragility in NF1 long bone dysplasia.

## Materials and Methods

### Mouse breeding and genotyping

Nf1Prx1 and Nf1Col1 mice were bred and genotyped as described previously [11], [13]. All experimental procedures were approved by the ‘Landesamt für Gesundheitsschutz und Technische Sicherheit (LaGeTSi), Berlin, Germany (protocol number ZH 120) and the Institutional Animal Care and Use Committee (IACUC) at the Vanderbilt University Medical Center (protocol number M/06/508).

### Histology

For histological analysis, adult limbs were fixed in buffered 4% PFA, dehydrated in subsequent ethanol steps and embedded in methyl methacrylate (MMA) according standard laboratory procedures [28]. For histological assessment, 5 µm plastic sections (RM2255, Leica, Germany) were stained with a combined von Kossa/Masson&Goldner or von Kossa/Toluidin procedure. Bright light microscopy was performed with an Olympus BX60 (Olympus, Japan). To analyze presence of acid bone matrix proteins, sections were stained with AgNOR [29]. Deposited acidic matrix proteins appear brown with AgNOR staining, while negative regions remain unstained. The collagenous bone matrix was assessed with bright or polarized light microscopy after picrosirius red staining [30], [31] (Leica DMRB, Germany). Linear polarization microscopy was performed with a Leica DMRB at which the polarization and analyst filters were set with 90° to each other. In bright light, picrosirius red stains all collagenous connective tissue (e.g. bone) red. By polarized microscopy, collagen fibers appear in shades of green, yellow and red. The polarization color indicates fiber thickness and degree of packaging [30], [31]. Red colored collagen fibers are highly packed and thick, while green colored fibers are less packed and disorganized [31]. Histomorphometric analysis was performed with AxioVision Software (Zeiss, Germany). Nomenclature was used according to ASBMR guidelines [32]. The ASBMR nomenclature does not provide a term for blood vessels, we used BlVes. Statistical analysis was performed with the unpaired t-test, * p≤0.05, ** p≤0.01.

### Immunohistology

Immunodetection of proteins within mineralized cortical bone was achieved according to standard procedure [33]. Frozen tissue was sectioned (5 µm) on a cryostat (Leica CM3050 S, Wetzlar, Germany). Sections were fixed in PBS buffered 4% PFA, then permeabilized with 0.1% saponin in PBS buffered 3% BSA, and blocked with 5% donkey serum. Antibodies were incubated in PBS buffered 3% BSA. The following antibodies were used for immunodetection: panendothelial cell antigen (550563, BD Pharmingen, USA) and anti-ratAlexa555 (A11081, Invitrogen, USA). Sections were analyzed with confocal microscopy (lsm 510, Zeiss, Germany).

### Micro computed tomography (microCT) analysis

Bone samples were analyzed with low (6 µm voxel) or high (up to 1 µm voxel) resolution microCT from skyscan 1172 (skyscan, Belgium). For BMD analysis, samples were measured with a multi-sample holder at a 6 µm pixel size, 180° degree rotation, rotation angle 0.3°, filter Al 0.5 mm, averaging 4, random movement 3, voltage 80 kV, and current 120 µA. Raw data reconstruction occurred with NRecon, with a defined threshold of 0.00 to 0.10, smoothing 3, ring artefact correction 9, and beam hardening correction 30%. Quantitative evaluation was performed with CTan after definition of appropriated regions of interest (ROIs). For analysis of cortical bone porosity, samples were measured as single samples at 1 µm pixel size, 180° degree rotation, rotation angle 0.3°, filter Al 0.5 mm, averaging 4, random movement 10, voltage 80 kV, and current 120 µA. Quantitative evaluation was performed with CTan within a ROI comprising 1300 slices. Nomenclature was used according ASBMR guidelines [32]. We used the terminology micro-porosity for Ot. lacunae (lacunae range from 100–4000 µm^3^) and macro-porosity (lacunae range 0.05–1*10^6^ µm^3^) for large non-mineralized structures. Volumes for large pores comprise volumes occupied by blood vessels and non-mineralized bone tissue. Statistical analysis was performed with the unpaired t-test, * p≤0.05, ** p≤0.01.

### Sample preparation for mechanical tensile measurements

Bone samples were sectioned from the mid-diaphysis of femora of three-month old mice and at other time points (Table S2). Femora were sectioned along the tangential-longitudinal plane. Each half of the femoral cortex was polished mechanically from both sides in an automatic polisher (Logitech PM5, Logitech Ltd., Glasgow, UK) until the sample was planar. Care was taken to make sure the samples’ longitudinal axes were parallel to the main longitudinal bone axis. Polishing was performed using sequentially 5, 3, and 1 µm grit-sized diamond particles (DP-Spray P, Struers A/S, Ballerup, Denmark) until the final sample thickness of 50 µm was achieved. Gross sectioning of cortical bones was performed with a slow speed saw (Isomet Low Speed Saw, Buehler Ltd., Lake Bluff, IL, USA) and further processed by UV laser micro-dissection, prior to spectroscopic and micro-mechanical probing of the tissues. The samples were further sectioned in an UV laser micro-dissection system (PALM MicroBeam C, P.A.L.M. Microlaser Technologies GmbH, Bernried, Germany) to isolate sections with the dimension of 150 µm width×50 µm thickness ×2.5 mm length. All sectioning and micro-dissection were performed on hydrated samples.

### Mechanical Tensile analysis

Tensile analysis was performed as described elsewhere [34]. Tensile experiments were performed with a custom-made micro-tensile testing apparatus with a translation motor (M-126.DG, Physik Instrumente, Karlsruhe, Germany) and a 250 g load cell (ALD-MINI-UTC-250, A.L. Design Inc., Buffalo, NY, USA) at a constant strain rate of 0.2 µm s^−1^ to simulate quasi-static loading. Each sectioned sample was glued to stiff teflon foils with cyanoacrylate (Loctite Deutschland GmbH, Munich, Germany) and mounted into the tensile apparatus so that the tensile axis was parallel to the longitudinal axis of the sample. Strain measurements were accomplished by measuring the percent displacement between optical marks made by a 50 µm tip sized marker (Copic Multiliner, Too Marker Products, Japan) on the sample and tracked by a video camera (Basler A101f, Basler Vision Technologies, Ahrensburg, Germany). Both the camera and the tensile tester were controlled by customized software (Labview 7.0, National Instruments). Each sample was tensed until fracture. Stress-strain behaviours were obtained for each sample measurement and further analysis were performed to determine characteristic material properties. Each data point tested was a mean average of three samples. All samples were kept hydrated during measurements and tensed until failure.

### Scanning acoustic microscopy (SAM)

SAM measurements were performed as described elsewhere [35]. Methylmethacrylate (MMA) embedded humeri were longitudinally sectioned to medial level, showing a complete open bone marrow cavity and separated anterior and posterior cortex. Subsequently, flat sample surfaces were prepared with a grinding procedure, using silicon carbide abrasive papers (grit size 4000; Phoenix 4000, Buehler, Düsseldorf, Germany). Afterwards the surface was polished with a hard synthetic cloth, ethyleneglycol suspension and 1 µm diamond particles. Samples were measured using a custom-made scanning acoustic microscope (SAM200Ex, Q-BAM, Halle, Germany) equipped with a spherically focused transducer with a nominal center frequency of 200 MHz (FHI 200/60°: Frauenhofer Institut für Biomedizinische Technik, St. Ingbert, Germany), providing a spatial resolution of approximately 8 µm in the focal plane. All measurements were performed in distilled and degassed water at 26±0.3°C. The sample surfaces were aligned in the focal plane of the transducer. A scan increment of 4 µm in x and y direction was chosen. For each (x,y) position the entire pulse echo signal V_x,y_(t) was stored. After the x,y scan, another x-scan with variable transducer-sample distance (z-scan) was performed for each sample within a bone area to perform a defocus correction. The confocal reflection amplitude was converted to values of the reflection coefficient *R* and acoustic impedance *Z* (in Mrayl; 1 rayl  = 1 kgm^−2^s^−1^), as described elsewhere [35]. The latter is equivalent to the square root of the product of mass density and apparent stiffness coefficient *c* [36]. For bone tissue, *Z* has been shown to be strongly correlated with the apparent stiffness (R^2^ = 0.996) [37], [38] and can therefore be used as a surrogate to assess variations of tissue stiffness.

### Small-angle X-ray scattering (SAXS) and energy dispersive X-ray spectroscopy (EDX)

SAXS measurements were performed as described elsewhere [39]. Longitudinal sections of MMA embedded humerus with a thickness in the order of the beam size (around 200 µm) were mounted on an automatic sample holder for polishing such that the longitudinal cross section is exposed at the surface. An X-ray beam with a wavelength of λ = 0.154 nm was generated using an X-ray generator (Bruker, AXS, Karlsruhe, Germany) with a rotating copper anode (40 kV/100 mA). The SAXS signal was measured with a 2D area detector (Bruker). Measurements were performed within cortical bone, following the bone cortex from proximal to distal in ∼400 µm step size. Two-dimensional SAXS patterns were evaluated for mean mineral particle thickness (T-parameter) and degree of particle alignment (Rho-parameter). The T-parameter measures the average thickness of mineral particles and higher values suggest increased volume of mineral platelets. The Rho-parameter indicates the relative orientation of mineral particles, where value “0” specifies no predominant orientation while “1” suggests parallel alignment.

EDX measurements were performed with a desktop scanning electron microscope (SEM) device (Hitachi TM-3000, Hitachi High-Technologies Europe GmbH, Krefeld Germany) on MMA embedded humeri that were prepared in a similar fashion to SAXS samples. Samples were mounted onto double sided carbon tape and subsequently, placed in the SEM sample holder and moved approximately until 1 mm away from the source of the primary beam. An accelerating voltage of 15 kV was used to examine the greyscale levels of the mineral content in the respective Nf1Prx1 and Nf1Col1 samples. An EDX unit (Bruker SAXS GmbH, Berlin Germany) was used to collect elemental spectra, specifically at energies corresponding to 2.0 and 3.7 keV, representing spectral signals for phosphorus and calcium, respectively. Whole EDX maps were created to map mineralization degree and element gradients in whole humeri tissues from respective Nf1Prx1 samples. Analysis of EDX images was performed with ImageJ (Rasband WS. ImageJ, U.S. National Institutes of Health, Bethesda, Maryland, USA, imagej.nih.gov/ij/, 1997–2012).

## Results

### Blood vessel associated mineralization defects destabilize Nf1Prx1 bone diaphysis

**Figure 1:**
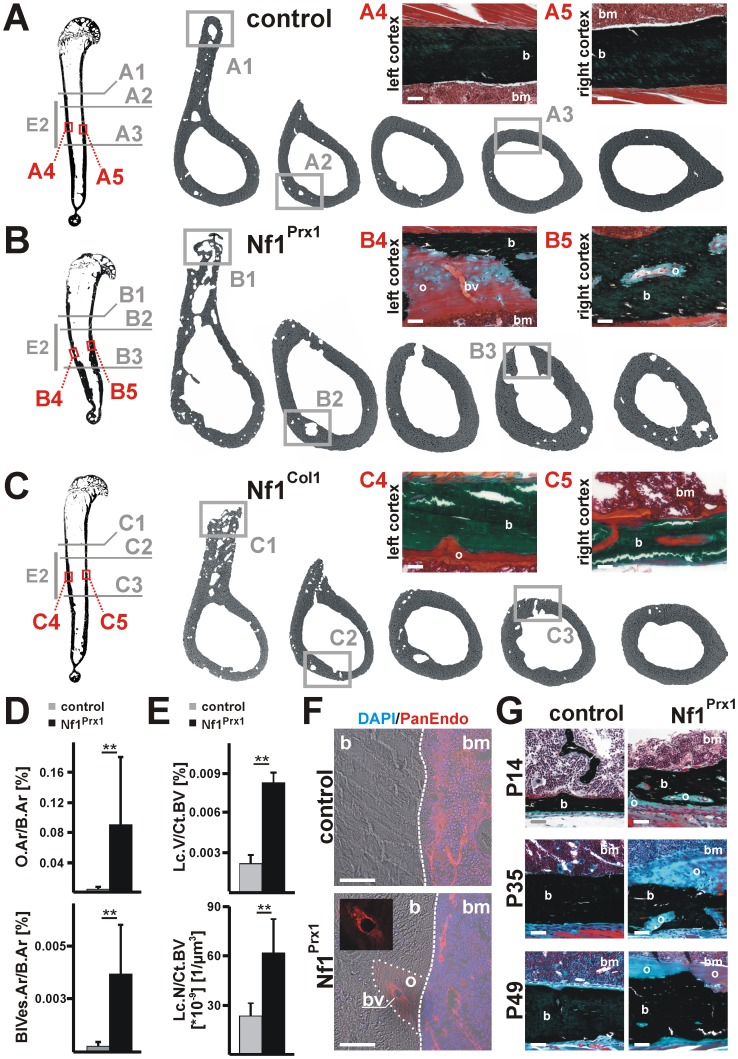
Nf1Prx1 mice show increased macro-porosity and ectopic blood vessels associated mineralization defects. (**A–C**) High-resolution micro-CT (skyscan) and von Kossa/Masson-Goldner histology of adult humeri. Consecutive cross-sections of the mid-shaft region (E2) in Nf1Prx1, Nf1Col1 and control mice representing following morphological sites: deltoid tuberosity (1), nutrient artery (2), bone cortex between deltoid tuberosity and epicondylus medialis (3), left cortex (4), and right cortex (5). (**A**) (A1) Deltoid tuberosity of controls showed solid appearance and smooth mineralization front. (A2) The site of nutrient artery bone cortex traverse. Note a fully mineralized, solid cortical bone in proximity of blood vessel. (A3, A4, A5) Diaphyseal cortical bone in control mice was free of macro-pores. (**B**) Morphology of Nf1Prx1 humeri. (B1) Note a broadened and inhomogeneously mineralized tuberosity with rough mineralization boundaries and increased porosity. (B2) A massively enlarged nutrient artery traversing site and (B3) a large ectopic lesions were observed in the cortical bone of Nf1Prx1 mice. (B4, B5) von Kossa/Masson Goldner histology of the diaphyseal bone cortex in blood vessel proximity. Note, unmineralized bone matrix (osteoid) surrounding the centrally localized blood vessel. (**C**) Nf1Col1 mouse humeri. (C1) Mutant deltoid tuberosity is widened with inhomogeneous mineral distribution and uneven bone boundaries. (C2-3) The size of the nutrient artery traversing site within diaphyseal bone cortex was normal in Nf1Col1 mice. (C4-5) In Nf1Col1 cortical bone small mineralization defects show a narrow osteoid rim surrounding blood vessels. These changes were of much smaller size compared to the Nf1Prx1 model. (**D**) Histomorphometric analysis of humerus cortical bone in the region E2 of Nf1Prx1 mice. Increased relative unmineralized bone tissue area (O.Ar/B.Ar) and blood vessel area (BlVes.Ar/B.Ar) (control n = 3, Nf1Prx1 n = 5). (**E**) Volume and number of mineralization defects (lacunae range 0.05–1*10^6^ µm^3^) in Nf1Prx1 cortical bone (region E2). High-resolution scans were evaluated with CTan (skyscan) software. Increased relative cortical bone porosity (summed lacunae volumes per cortical bone volume, Lc.V/Ct.BV) as well as lacunae number (Lc.N/Ct.BV) (control and Nf1Prx1 n = 5) in Nf1Prx1 humeri. Statistical significance - t-test, ** p≤0.001. (**F**) Immunostaining with pan-endothelial antibody confirms presence of blood vessels within mineralization defects in Nf1Prx1 cortical bone. (**G**) Nf1Prx1 cortical bone in the region E2, von Kossa/Masson-Goldner histology showing mineralization defects through developmental stages P14, P35 and P49. Scale bars - 50 µm. Abbreviations: blood vessels (bv), bone (b), bone marrow (bm), osteoid (o).

**Figure 2:**
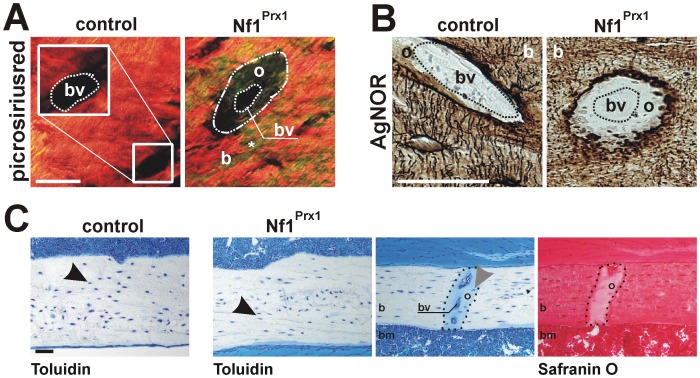
Diminished organic matrix properties in Nf1Prx1 mice. (**A**) Picrosirius red stained bone sections imaged with polarized light. Homogenous red staining in controls indicates highly packed and thick collagen. Heterogeneous red-yellow-green staining of Nf1Prx1 bone sections is indicative of diminished packaging and thickness of bone collagen. Note, there is no picrosirius red staining within non-mineralized bone tissue (osteoid) surrounding blood vessels. Scale bar shows 20 µm. (**B**) Silver staining (AgNOR) detects the osteocytic network and other accumulations of acidic matrix proteins. Note the large unstained area around blood vessels in Nf1Prx1 humerus. Scale bar shows 50 µm. (**C**) Sections of humerus cortex stained with Toluidin and Safranin O. Toluidin stained osteocyte (Ot.) appear blue with dark blue nuclei (black arrowhead). Presence of nuclei suggests vitality of cells in the bone cortex. Osteoid (dotted line) near to the blood vessel was stained light blue (Toluidin) or light red (Safranin O). Light blue or red staining indicates that bone lesions are not composed of cartilaginous matrix, which with these methods stains purple (Toluidin) or dark red (Safranin O) (not shown). Ot. in non-mineralized areas are viable (nuclei marked with grey arrow head). Scale bar represents 50 µm. Abbreviations: blood vessels (bv), bone (b), bone marrow (bm), osteoid (o).

Long bones of Nf1Prx1 mice show significant shortening, bowing and reduced trabecular bone mass [11]. To get insights into the cortical bone quality, we analyzed diaphyseal bone morphology at three months of age with high resolution micro-computed tomography (microCT). Since hind limb movement in Nf1Prx1 mice is compromised due to a hip joint fusion, we focused our analysis on the humerus. Control humeri had a straight shape with a pronounced *tuberositas deltoideus* (A1), a distinct *arteria nutriens* traversing channel (A2) and solid bone cortex (A3) (Fig. 1A). By histology, the left and right bone cortex (A4, A5) appeared fully mineralized and rarely populated by blood vessels (Fig. 1A). In Nf1Prx1 mice microCT scans revealed enlarged and porous *tuberositas deltoideus* (B1) compared to controls (Fig. 1B). The main artery *arteria nutriens* (B2), which supplies blood to the bone marrow cavity, was strikingly enlarged in Nf1Prx1 mutants (Fig. 2B). Additionally, massive cortical bone defects (B3) were present in the distal humerus (Fig. 2B) of Nf1Prx1 mice that were absent in controls. Histological analysis (von Kossa/Masson&Goldner) demonstrates that these bone lesions were in fact regions of non-mineralized bone matrix (osteoid) adjacent to ectopic blood vessels (B4, B5) (Fig. 2B). In Nf1Col1 mice, characterized by *Nf1* inactivation in osteoblasts, the *tuberositas deltoideus* was enlarged and irregularly shaped (C1); however, the *arteria nutriens* had normal size (C2) (Fig. 2C). Fewer and smaller non-mineralized areas were observed in Nf1Col1 mice in the region where large demineralization spots were present in Nf1Prx1 humeri (C3, C4, C5) (Fig. 2C). Next, we quantified macro-porosities using histological and microCT methods. The relative osteoid area (O.Ar/B.Ar) and relative blood vessel area (BlVes.Ar/B.Ar) per bone area were increased in Nf1Prx1 mice by 25- and 12-fold, respectively (O.Ar/B.Ar: ctrl  = 0.0035±0.0026%; Nf1Prx1  = 0.0908±0.1254%; BlVes.Ar/B.Ar: ctrl  = 0.0003±0.0003%; Nf1Prx1  = 0.0037±0.0028%), in the ROI E2 (Fig. 1D). Quantitative microCT analysis corroborated these results. Both the relative summed lacunae volume (Lc.V/Ct.BV) and the relative lacunae number (Lc.N/Ct.BV) per cortical bone volume were increased (Lc.V/Ct.BV: ctrl  = 0.0022±0.0006; Nf1Prx1  = 0.0079±0.0011, Lc.N/Ct.BV: ctrl  = 23.0±8.0*10^−9^ n/µm^3^; Nf1Prx1  = 62.0±21.0*10^−9^ n/µm^3^) (Fig. 1E; Table S1). In contrast, no significant increase in blood vessel related bone porosity was observed in Nf1Col1 mice (Lc.V/Ct.BV: ctrl  = 0.0039±0.0003; Nf1Col1  = 0.0041±0.0017; Lc.N/Ct.BV: ctrl  = 28.2±7.3*10^−9^ n/µm^3^; Nf1Col1  = 36.2±13.4*10^−9^ n/µm^3^) (Table S1).

We confirmed the vascular endothelial identity of the cells within macro-porotic bone defects in Nf1Prx1 mice using immune-staining against pan-endothelial antigen (Fig. 1F). Additionally, vessel associated bone lesions were detected in humerus sections from all analyzed stages (P14, P35 and P49), suggesting a developmental origin of the phenotype (Fig. 1G). In addition, significant presence of these lesions in Nf1Prx1 mice (limb knock-out) but less voluminous in Nf1Col1 mice (osteoblast knock-out) suggests an endothelial lineage involvement in the pathogenesis of these changes.

### Reduced thickness and packaging of collagen in the proximity of diaphyseal blood vessels

Organic matrix appearance in blood vessel proximity was analyzed with picrosirus red and AgNOR histology. Similar to other matrix demineralization areas in *Nf1* deficient bones, the matrix near blood vessels stained pale-green with picrosirus red in polarized light indicating presence of collagen with reduced thickness as well as packaging (Fig. 2A). The AgNOR method gave intensive staining at the mineralization front, surrounding blood vessels, but the mineral-free zone in direct contact with the vessel was not stained (Fig. 2B). A possible cartilaginous nature of this organic matrix was excluded by Toluidin and Safranin O histology (Fig. 2C). Moreover, Toluidin and Safranin O staining demonstrated viability of osteocytes within mineralized and non-mineralized bone tissue. In summary, vessel-associated diaphyseal bone lesions contain collagen with reduced thickness and packaging that may, due to their large volumes, weaken cortical bone stability.

### Micro-dissected slices of NfPrx1 bone tissue are mechanically fragile

**Figure 3:**
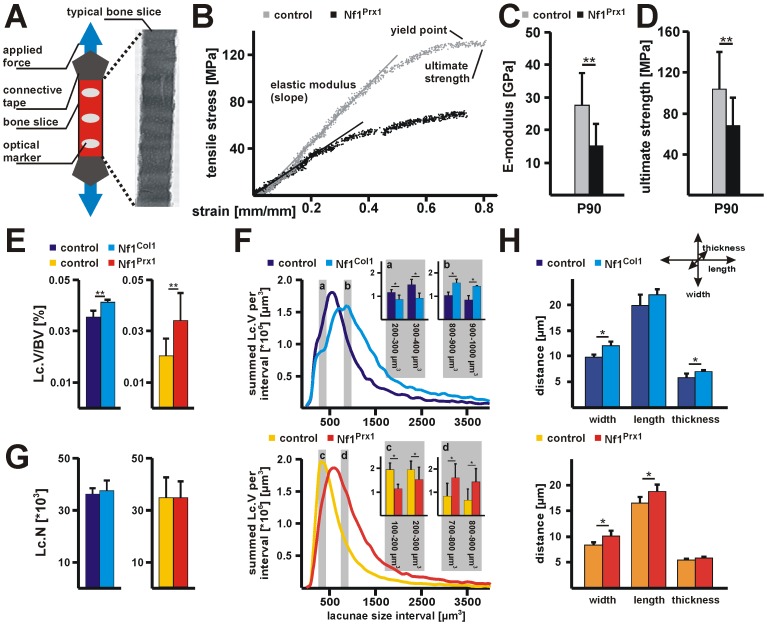
Nf1Prx1 bone tissue shows diminished mechanical strength and increased micro-porosity due to increased osteocyte lacuna size. (A) Diagram of the analytical setup used for tensile experiment. Measurements were done on laser dissected cortical bone slices from P90 mice. (B) A typical tensile stress-strain pattern in controls showed a linear elastic modulus (E-modulus) phase that is followed by the yield point plateau and the ultimate stress point. Note, increased elasticity and diminished ultimate strength in bone slices of Nf1Prx1 humerus compared to controls. (C) Diminished E-modulus (slope of tensile curve) and (D) ultimate stress point (point of maximal load before tissue failure) in bone tissue slices from 3 months old Nf1Prx1 (n = 14) and control (n = 15) mice. (E) Quantitative microCT analysis of the total (summed) lacunae porosity (Lc.V/BV) within the lacunae range from 100-4000 µm^3^. Note the significant increase of Lc.V/BV in Nf1Col1 and Nf1Prx1 mice. (F) The summed volume of Ot. lacunae (Lc.V) within the size range 100–4000 µm^3^ with an increment of 100 µm^3^ (histogram). Note the shift of summed Lc.V fractions towards higher volume range in Nf1Col1 and Nf1Prx1 mutants. Insert (a) - Nf1Col1 mice demonstrated reduced summed Lc. volume in the intervals 200–300 and 300–400 µm^3^ and (b) - summed Lc.V in fractions 800–900 and 900–1000 µm^3^. Insert (c) - Nf1Prx1 mice showed reduced summed Lc. volume in the intervals 100–200 and 200–300 µm^3^ and (d) - summed Lc.V in fractions 700–800 and 800–900 µm^3^. (G) The total number of Ot. lacunae (Lc.N) was unaffected in both mouse models. (H) Ot. lacunae morphology (range from 100–4000 µm^3^) assessed by measuring the x, y and z axis length in a selected volume of ROI E2. Note, lacunae size is increased in all dimensions in Nf1Col1 and Nf1Prx1 mice. The number of analyzed bone samples: Nf1Prx1 n = 5, controls n = 5; Nf1Col1 n = 3, controls n = 3. Statistical analysis was performed with unpaired Student t-test, * p≤0.05 ** p≤0.01.

Since large matrix mineralization defects in the Nf1Prx1 diaphysis were local, we asked if micro-scale properties of the mineralized bone tissue were also altered. In order to measure mechanical strength of the bone material, we performed tensile analysis on bone tissue slices obtained by laser micro-dissection (Fig. 3A). Typical tensile test traces are composed of three phases, the elastic modulus, yield point, and ultimate strength. The linear slope gives the elastic modulus (Young's or E-modulus), the yield point is where the stress-strain curve levels off and inelastic sample deformation begins to occur and the ultimate strength is obtained from the stress point where the bone material ruptures (Fig. 3B). Bone tissue slices from adult Nf1Prx1 humeri showed a 50% reduction of E-modulus (ctrl  = 27.5±9.9 GPa; Nf1Prx1  = 15.0±6.7 GPa) and 35% decrease of ultimate strength compared to controls (ctrl  = 103.9±35.8 MPa; Nf1Prx1  = 67.8±27.5 MPa) (Fig. 3C–D). A similar reduction of the bone material mechanical strength was observed in samples from other developmental stages (P4, P18, P60, P120) (Table S2). Although cortical bone slices with large defects were excluded from the analysis, mutant bone slices often contained microscopically visible large porosities that were not observed in controls (data not shown). The increased macro-porosity could explain mechanical instability. However, other factors including micro-porosity, degree of bone tissue mineralization and organic matrix quality are also likely to be involved [16], [17], [23], [24], [25], [26].

### Increased micro-porosity in Nf1Prx1 and Nf1Col1 bone tissue due to osteocyte lacunae enlargement

To understand the cause of decreased bone material mechanical resistance, we analyzed osteocyte (Ot.) lacunae size and number in Nf1Prx1 and Nf1Col1 humeri using high resolution microCT. We analyzed cortical bone within the diaphyseal ROI E2 (Fig. 1A). The summed Ot. lacunae volume per bone volume (Lc.V/BV) was almost doubled in Nf1Prx1 and significantly increased in Nf1Col1 mice compared to respective controls (level E2: ctrl  = 0.020±0.007%, Nf1Prx1  = 0.034±0.011%; ctrl  = 0.036±0.003%, Nf1Col1  = 0.041±0.002%) (Fig. 3E, Table S1). Lacunae volume distribution (the summed lacunae volume within a 100 µm^3^ size interval) was shifted toward larger size intervals within Nf1Prx1 and Nf1Col1 mutant diaphysis (Fig. 3F). Nf1Prx1 mutants showed a peak maximum at 600 µm^3^ whereas control samples had a peak maximum at 300 µm^3^. Statistical significance of these changes was illustrated for the selected size intervals 100–300 µm^3^ and 700–900 µm^3^ (Fig. 3F, insets c and d). Similar changes of lacunae volume distribution were observed in Nf1Col1 bones (Fig. 3F, insets a and b). In contrast, relative Ot. number (Ot.N) per bone tissue volume was not altered in the Nf1Prx1 humerus (Fig. 3G). We further assessed lacunae morphology by measuring the x, y and z dimensions. In both mouse models, Nf1Prx1 and Nf1Col1, osteocyte lacunae morphology was enlarged in all measured dimensions (Fig. 3H). Collectively these data indicate that Ot. viability is not impaired by *Nf1* inactivation in bone, but causes increased osteocyte volume and abnormal morphology.

### Global defect of bone matrix formation and bone mineral content in *Nf1* deficient cortical bone

**Figure 4:**
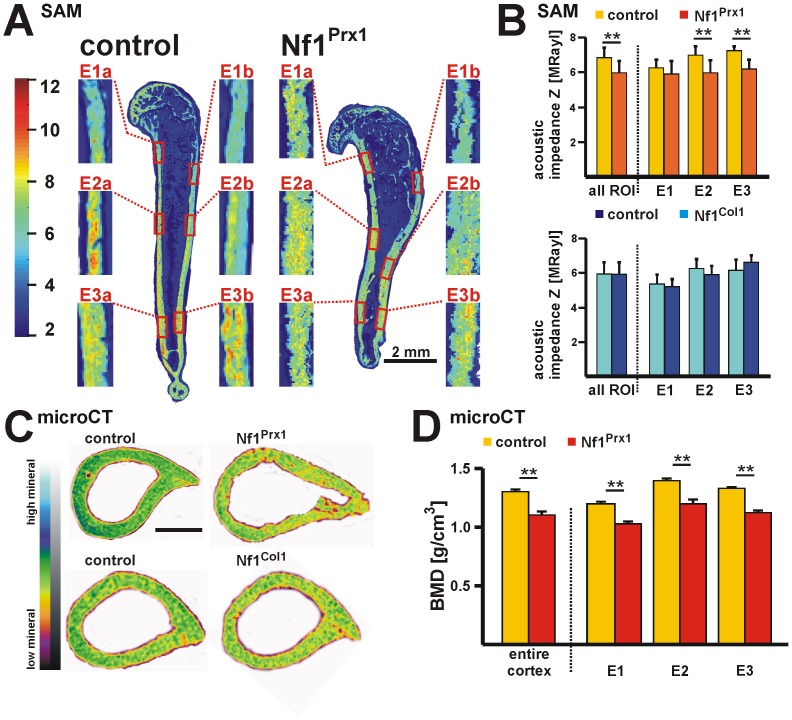
Diminished bone stiffness, bone mineralization and organic matrix formation in Nf1Prx1 mice. (**A**) Scanning acoustic microscopy (SAM) was applied to show elastic properties of the bone matrix. SAM measurements were performed within six ROIs E1a–E3b on anterior (a) and posterior (b) bone cortex. Images illustrate impedance values (Z in MRayl) according to the color scale (left). Nf1Prx1 mutants show diminished impedance values within all analyzed ROIs. (**B**) Quantitative evaluation of SAM measurements reveals decreased Z values in Nf1Prx1 humerus in all analyzed ROIs (control n = 4, Nf1Prx1 n = 4). Nf1Col1 humeri did not demonstrate diminished Z values (control n = 5, Nf1Col1 n = 4). (**C**) The cross sections of humerus at the level E2 with color coded BMD values measured by phantom calibrated microCT. The yellow and green coloring indicates low and high BMD values, respectively. Scale bar - 600 µm. (**D**) Cortical bone mineral density (BMD) was assessed by microCT within region E1–E3 in humeri of Nf1Prx1 (control n = 5, Nf1Prx1 n = 5) mice. BMD was reduced in the Nf1Prx1 model in all ROIs (for definition see Fig.S1). Statistical significance - t-test, * p≤0.05 and ** p≤0.01.

To further characterize bone matrix quality in the *Nf1* deficient humerus cortex, we performed acoustic impedance measurements by scanning acoustic microscopy (SAM). High (red) acoustic impedance values (Z) indicate high stiffness and low Z-values (greenish/blue) soft material properties (Fig. 4A). Selected regions represent the heterogeneity of matrix stiffness throughout the entire humerus bone cortex. Quantitative analysis was performed within six ROIs in anterior and posterior bone cortex (E1–E3). Z-values were heterogeneous throughout humerus cortex length (Fig. 4B). Nf1Prx1 mutant bones had reduced Z-values in ROIs E2 and E3 indicating softening of the mineralized organic matrix in the mid shaft humerus (average Z of all ROI: ctrl  = 6.83±0.59 MRayl; Nf1Prx1  = 5.96±0.70 MRayl) (Fig. 4B). However, in Nf1Col1 mice the acoustic impedance was unaffected (average Z of all ROI: ctrl  = 5.92±0.69 MRayl; Nf1Col1  = 5.90±0.73 MRayl) (Fig. 4B, Table S3). These results indicate that loss of neurofibromin induces impairment of the bone matrix stiffness that is most pronounced in the bowed part of Nf1Prx1 bones but not in Nf1Col1 bones.

Phantom calibrated microCT was used to assess bone mineral density (BMD) throughout cortical bone in Nf1Prx1 and Nf1Col1 humerus (Fig.S1A). Significant reduction of BMD was detected in Nf1Prx1 (ctrl  = 1.299±0.020 g/cm^3^, Nf1Prx1  = 1.102±0.027 g/cm^3^) and Nf1Col1 mice (ctrl  = 1.369±0.007 g/cm^3^, Nf1Col1  = 1.271±0.024 g/cm^3^) (Fig. 4C–D, Fig.S1A). Similarly to changes in Ot. lacuna size, the degree of BMD reduction was higher in the Nf1Prx1 mice than in the Nf1Col1 model. Low bone mineral content in Nf1Prx1 humerus was confirmed by energy dispersive X-ray spectroscopy (EDX) on three ROIs (E1–E3) (Fig.S1B). The calcium to phosphorus ratio was unaffected in Nf1Prx1 humerus compared to controls (ctrl  = 1.9680±0.0772, Nf1Prx1  = 1.9670±0.0636). To obtain information about mineral particle size and orientation, small-angle X-ray scattering (SAXS) imaging was applied. The mean mineral particle thickness and the degree of particle alignment were similar in mutant and control samples (Fig.S1C–D). Thus, *Nf1* deficiency causes low bone mineral density without affecting mineral particle size and orientation.

### Bone tissue from individuals with NF1 and tibial dysplasia shows inhomogeneous mineralization, increased micro-porosity and diminished collagen fiber thickness

**Figure 5:**
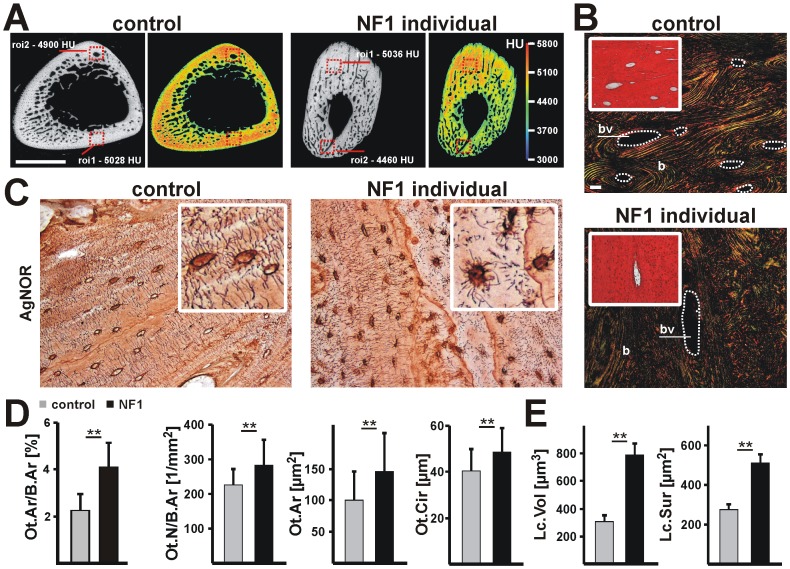
Cortical bone in NF1 tibial dysplasia is characterized by heterogeneous mineralization, diminished collagen thickness and increased micro-porosity. (A) MicroCT analysis of human cortical bone samples (tibia). Bone mineral density was calibrated according to Houndsfield units (HU) and color coded (see HU scale). HU values measured in ROI1 (cortical bone without blood vessels) and ROI2 (cortical bone near blood vessel) were in control specimen similar. However, in the NF1 bone sample HU values were in proximity of large vessels decreased (ROI2). The analyzed bone sample was obtained pre-fracture from an individual affected by NF1 with tibial bowing. (B) Two further mineralized bone samples were obtained from cortical bone of NF1 individuals with pseudarthrosis. Samples were analyzed by picrosirius red histology and imaged in polarized light (large image) and bright light (inset). Collagen fibers follow osteonal organization in control specimens. Note that in the NF1 cortical bone sample collagen fiber organization appears less orderly with abundant thin (green) collagen fibers. (C) Ot. morphology was visualised with AgNOR staining. Ot. are spindle shaped (inset) and regularly distributed in control cortical bone. In contrast, Ot. are round and irregularly distributed in NF1 cortical bone. (D) Histomorphometry of AgNOR stained bone sections showing: relative Ot. area (Ot.Ar/B.Ar), Ot. number per bone area (Ot.N/B.Ar), individual Ot. area (Ot.Ar) and individual Ot. circumference (Ot.Cir). Presented data are mean values with standard deviations (control n = 3, NF1 n = 2). (E) Volumetric microCT analysis showing increased specific lacunae (Ot.) volume (Lc.Vol) and surface (Lc.Sur) in NF1 tibial dysplasia cortical bone as compared to controls. Statistical analysis was performed with unpaired t-test, ** p≤0.01. Abbreviations: blood vessels (bv) and bone (b). All scale bars represent 50 µm.

**Figure 6:**
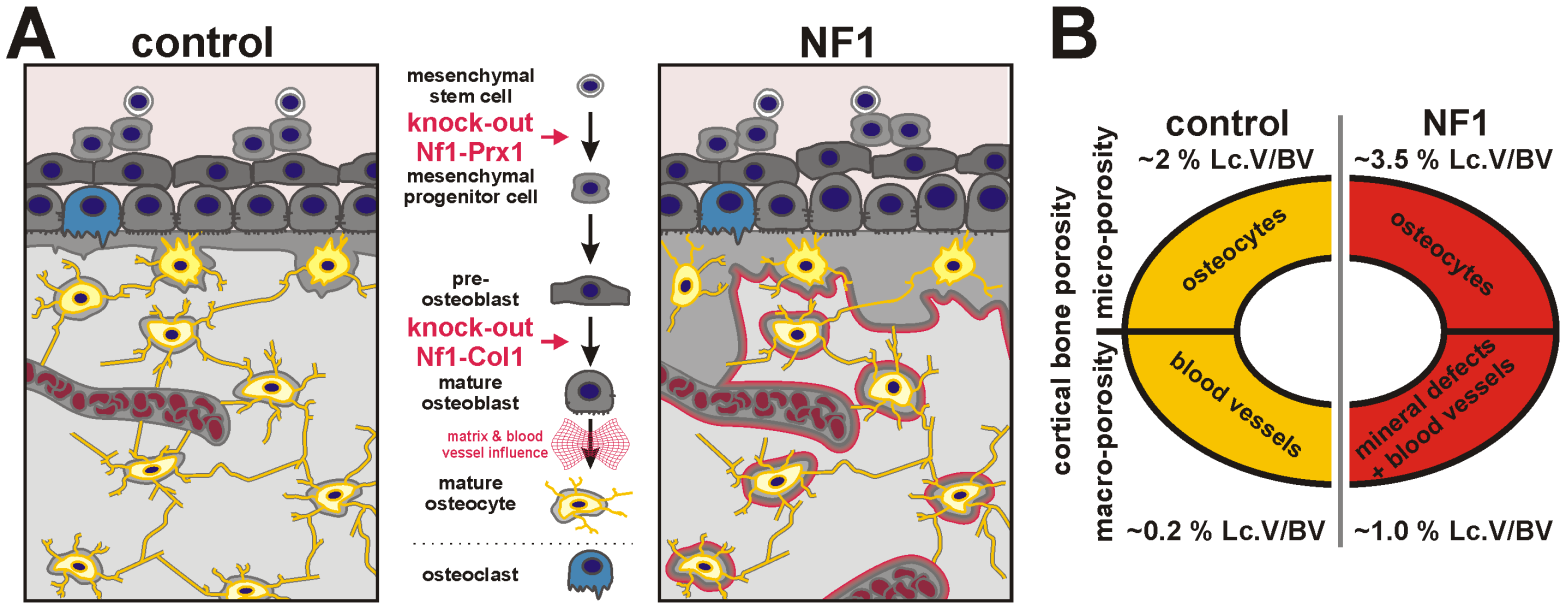
Neurofibromin is a critical regulator of cortical bone integrity and function. (**A**) Ablation of *Nf1* in pre-osteoblasts (Nf1Col1) results in low bone mass phenotype with hyperosteoidosis. Loss of *Nf1* in mesenchymal progenitor cells (Nf1Prx1) produces a complex phenotype characterized by low bone mass, hyperosteoidosis, increased micro-porosity (Ot.), macro-porotic mineralization lesions, and persistence of blood vessels. Moreover, loss of neurofibromin causes defective inorganic and organic bone matrix formation especially in proximity of blood vessels. (**B**) Micro- and macro-porosity contributes differentially to overall structural destabilisation in NF1 bone. In controls micro- (Ot.) and macro-porosity (blood vessels) encompasses approx. 2% and 0.2% of cortical bone volume, respectively. In Nf1Prx1 mutant bone micro-porosity is elevated to approx. 3.5%. Importantly macro-porosity is amplified to 1.0% due to blood vessel persistence and bone mineral lesions.

We analyzed human cortical bone samples that were surgically removed from three individuals with NF1 tibial dysplasia. The samples included a fracture/pseudarthrosis lesion and a cortical bone sample removed pre-fracture. Age matched cortical bone samples were used as controls. Low-resolution microCT analysis of a pre-fracture NF1 tibial dysplasia sample showed thickening of the tibia bone cortex and increased macro-porosity (Fig. 5A). In the control bone sample, bone mineral density (BMD) distribution was homogeneous as revealed by even yellow/red coloring (Hounsfield unit [HU] calibration) and similar BMD values were measured at two distant sites within the bone shaft (ROI1 5028 and ROI2 4900 HU). In contrast, the NF1 cortical bone sample showed unbalanced mineral distribution with a BMD decrease of approx. 20% between ROI1 (5036 HU) and ROI2 (4460 HU). Picrosirius red stained control bone sections appeared red and yellow under polarized light, which is indicative of highly ordered and thick collagen fibers that followed circular osteonal organization (Fig. 5B). Two further surgically removed bone samples from individuals with NF1 tibial dysplasia, originating from cortical bone adjacent to a pseudarthrotic bone lesion, were analyzed histologically and with microCT. Picrosirius red stained NF1 bone biopsies stained in shades of orange, yellow and green, suggesting presence of less thick and packed collagen fibers. The overall Ot. morphology in NF1 bone samples analyzed by the AgNOR method appeared more heterogeneous and their lacunae size was increased (Fig. 5C). Histomorphometry revealed increased relative Ot. occupied area (Ot.Ar/B.Ar) in NF1 biopsies compared to controls (ctrl 2.25±0.72%; NF1 4.12±1.00%) (control n = 3, NF1 n = 2; analyzed cell number >1500 each group) (Fig. 5D). We also detected a statistically significant increase of Ot. number (Ot.N), individual Ot. area (Ot.Ar) and individual Ot. circumference (Ot.Cir) in NF1 bone samples. Accordingly, volumetric microCT analysis revealed increased Ot. lacunae volume (Lc.Vol) and Ot. surface (Lc.Sur) in NF1 bone samples (Fig. 5E). Thus, NF1 tibial dysplasia cortical bone samples show heterogeneous mineral distribution, poor collagen fiber thickness and increased micro-porosity, which is similar to changes in Nf1Prx1 and Nf1Col1 mice (Fig. 6A–B).

## Discussion

Our analysis indicates that multiple changes contribute to the reduction of bone mechanical strength upon loss of neurofibromin. Previous studies documented dysregulated osteoblast and osteoclast activity in *Nf1* deficient bone, yielding a high-turnover phenotype, reduced bone mass and low bone mineral content [11], [13], [14], [15]. We now show that *Nf1* inactivation affects osteoblasts and osteocytes to various extents, depending on the localization in the cortical bone, with the most dramatic effects seen in the proximity of blood vessels. In blood vessel regions, osteoblast and osteocyte function is compromised, triggering formation of local osteomalatic lesions (Fig. 6A–B).

Based on the localization of these changes within the long bone diaphysis and general long bone bowing, we postulate that one of the factors exacerbating matrix mineralization defects in NF1 is mechanical strain. Mechanical stimuli are required for normal osteoblast and bone development [40]. Upon *Nf1* ablation, diaphyseal accumulation of defective organic bone matrix is most likely triggered by mechanical loads on an already poor bone cortex structure. Mechanical stimulation has been recently shown to induce ERK activation and osteoblast proliferation through integrins [41]. It has also been shown that FGFs control expression of matrix mineralization genes essential for osteocyte differentiation through ERK signaling [41]. Consequently, various aspects of the bone phenotype in Nf1Prx1 mice are linked to hyperactivation of the MAPK pathway throughout bone ontogeny [11], [13], [28], [42]. The net outcome of MAPK signaling in bone appears to be dependent on signal intensity as well as the stage of osteoblast differentiation, with an inhibitory effect in early osteoblasts [43], [44], and pro-osteogenic effect in mature osteoblasts [45]. In support of this notion, *Nf1* ablation in early osteoblasts causes decreased matrix mineralization, while inactivation in late osteoblasts has opposite effects [46]. Thus, adequate intensity and timing of MAPK activation are prerequisite for normal bone formation [47].

Loss of *Nf1* has a pro-osteogenic effect in mature osteoblasts in the absence of excessive mechanical stimulation or blood vessel influences. This is strikingly different in areas of high mechanical load (diaphysis) as well as in proximity of blood vessels. In diaphyseal cortical bone, the loss of *Nf1* results in catastrophic deterioration of organic matrix quality and mineralization, resulting in local osteolysis/osteomalacia. Perhaps the most important observation of this study is the discovery of collagen with reduced thickness and packaging in proximity of ectopic blood vessels. Blood vessel-associated diaphyseal defects (macro-porosities) by proportion, contribute the most to the overall reduction of Nf1Prx1 bone structural stability (Fig. 6B). The size of these lesions and localization within the mid-shaft of bone, where the torsion and longitudinal forces coalesce, seems to pre-determine potential fracture sites. We have previously shown that *Nf1* excision rates in endothelial cells isolated from Nf1Prx1 tissue were high [11]. Thus, we hypothesize that the consequent deregulation of signaling in endothelial cells contributes to the vessel-associated bone anomalies.

Another factor involved in the etiology of macro-porotic lesions in the Nf1Prx1 model is a complete absence of the acidic matrix proteins, which mediate mineral organization in the organic collagenous matrix. This is intriguing, as acidic matrix proteins are critical for mineral binding and organization. For instance, the acidic matrix protein Dmp1 binds to collagen and facilitates site-specific HA mineral deposition and controls collagen assembly *in vitro* [48], [49]. An important question to answer in future studies will be the cause of acidic matrix protein exclusion from ECM in vessel proximity. It appears that changes in the properties of *Nf1*-deficient vessels contribute to the bone demineralization. This might be caused by altered blood vessel barrier and diffusion properties [50] allowing blood serum factors with matrix mineralization inhibitory properties to access the bone matrix, e.g., fetuin-A [51]. While such a hypothesis awaits future experimental verification, some of the structural defects in cortical bone in Nf1Prx1 mice and in individuals with NF1 and tibial dysplasia develop at the interface between *Nf1* deficient bone cells and endothelium.

Structural analysis revealed increased micro-porosity in Nf1Col1 and Nf1Prx1 cortical bones was associated with increased osteocyte lacunae size, but not osteocyte number. Absence of non-nucleated lacunae indicates normal osteocyte viability, which is also supported by absence of augmented cortical bone remodeling as usually observed upon osteocyte ablation [52]. However, osteocytes in Nf1Prx1 humerus and NF1 bone tissue occupy larger bone volumes, indicating defective osteoblast-to-osteocyte differentiation and altered function (Fig. 6A–B). Together with defective organic matrix formation and mineralization, increased micro-porosity contributes to the severe mechanical impairment of Nf1Prx1 bone tissue [24], [26]. In light of the macro-porotic bone lesions, it is likely that increased micro-porosity is not the major determinant of bone fragility in NF1. However, interplay between osteocytes and endothelial cells leading to severe local bone lesions in sites of high mechanical force integration is possible.

On the nano-scale level, bone strength is determined by the collagen primary and secondary structure, tropocollagen assembly, formation of mineralized collagen fibrils, organization into fibril arrays, higher order patterning of collagen fibers, and lastly by mineral phase apposition [18], [53]. Disturbance at any one of these stages might perturb bone function. The complete lack of collagen polarization within macro-porotic bone lesions in Nf1Prx1 mice is a key observation, suggesting a critical role of neurofibromin for collagen formation. This is supported by irregular picrosirius red polarization in mineralized bone tissue and a reduced stiffness measured by SAM. The tensile analysis showed a ∼50% reduction of the E-modulus in Nf1Prx1 bone tissue, with ∼10% decreased impedance values of the bone matrix by SAM. Additionally, in adult Nf1Prx1 mice a 10% reduction of mineralization was present. However, it is important to note that overall mineral orientation measured by SAXS, providing an indirect measure of collagen orientation, is unaffected. Thus, we conclude that grossly normal collagen coexists with regions of diminished collagen thickness and packaging. Together, these findings suggest that *Nf1* ablation causes a primary defect of collagen formation. However, whether this directly affects mineralization remains speculative.

Little attention has been given to the mechanisms of blood vessel invasion during early development and their subsequent exclusion from the long bone cortex in postnatal development [21], [22]. Our data indicate that neurofibromin contributes to this process. Persistence of ectopic vessels together with collagenous matrix formation defects due to osteoblast dysfunction appears to drive the demineralization processes. Similar focal mineralization defects are present in cortical bone samples from individuals with NF1 and tibial dysplasia, both post-fracture and pre-fracture (one case). In these samples, we detected inhomogeneous cortical mineralization with low BMD in proximity of large vessels penetrating the bone cortex and enlarged osteocytes. Thus, increased osteocyte micro-porosity and vessel related macro-porosity are two major components of NF1 bone dysplasia pathology.

Although major observations made in Nf1Prx1 mice could be verified in human samples, our study is restricted by the limited number of analyzed NF1 patient bone samples and by the significant differences in bone organization between mouse and man. In contrast to mouse bone, human bone is supplied with blood by a canalicular system formed by Haversian and Volkmann's canals, resulting in osteonal bone organization and remodeling [17]. In human bone, collagen fibers are circularly aligned following osteonal organization. Thus, comparison of these two bone types is not straight forward. Further limitation of this study is that it does not address whether loss of neurofibromin is also associated with changes of biochemical bone tissue composition and/or alterations of collagen fiber morphology. This kind of analysis should be addressed in further studies [17], [18], [54], which will be instructed by the findings that loss of neurofibromin induces generalized defects of bone tissue function as well as highly localized structural alterations [11], [13], [15], [28].

## Supporting Information

Figure S1 **Small-angle X-ray scattering (SAXS) analysis demonstrates normal overall mineral orientation in Nf1Prx1 humerus.** (**A**) Cortical bone mineral density (BMD) was assessed by microCT within region E1–E3 in humeri of Nf1Col1 (control n = 3, Nf1Prx1 n = 3) mice. BMD was reduced in the Nf1Col1 model in all ROIs. ROIs E1, E2 and E3 were choosen as indicated. (**B**) Relative mineral content was imaged by energy dispersive X-ray spectroscopy (EDX) and mean grey value intensities were measured with AxioVision (Zeiss) (controls n = 3, mutants n = 3). All cortical regions E1–E3 of Nf1Prx1 mice showed lower degree of mineralization. (**C**) SAXS analysis revealed normal mineral particle thickness (T-parameter) within the midshaft region of control (grey) and Nf1Prx1 (black) humeri. Analyzed samples were from different postnatal stage P15, P42 and P90. (**D**) The mineral particle orientation (Rho-parameter) appeared also unaffected between controls and Nf1Prx1 humerus. Position of measured points according to the distance from the growth plate is indicated at the abscissa and the vertical lines indicate centre of humerus cortex. For each developmental stage, one humerus was analyzed. Horizontal lines and adjacent numbers represent the mean value of appropriated individual measurements. Measurements at different developmental time points showed no differences between control and Nf1Prx1 humeri for the T- or Rho-parameter suggesting normal mineral particle thickness and alignment.
(TIF)

Table S1 **Cortical volume parameter in P90 humeri level E2 of control, Nf1-Prx1 and Nf1-Col1 mice measured by high resolution micro-CT.**
(DOC)

Table S2 **Mechanical testing of control and Nf1-Prx1 humerus measured by tensile analysis.**
(DOC)

Table S3 **Acoustic impedance measurement of P90 humeri of control, Nf1-Prx1 and Nf1-Col1 mice measured by ultrasound microscopy.**
(DOC)
