# Supplementary figures and images for: Multiscale, Converging Defects of Macro-Porosity, Microstructure and Matrix Mineralization Impact Long Bone Fragility in NF1
Source: PLoS One. 2014 Jan 21;9(1):e86115. doi: 10.1371/journal.pone.0086115 (PMC3897656; doi:10.1371/journal.pone.0086115)

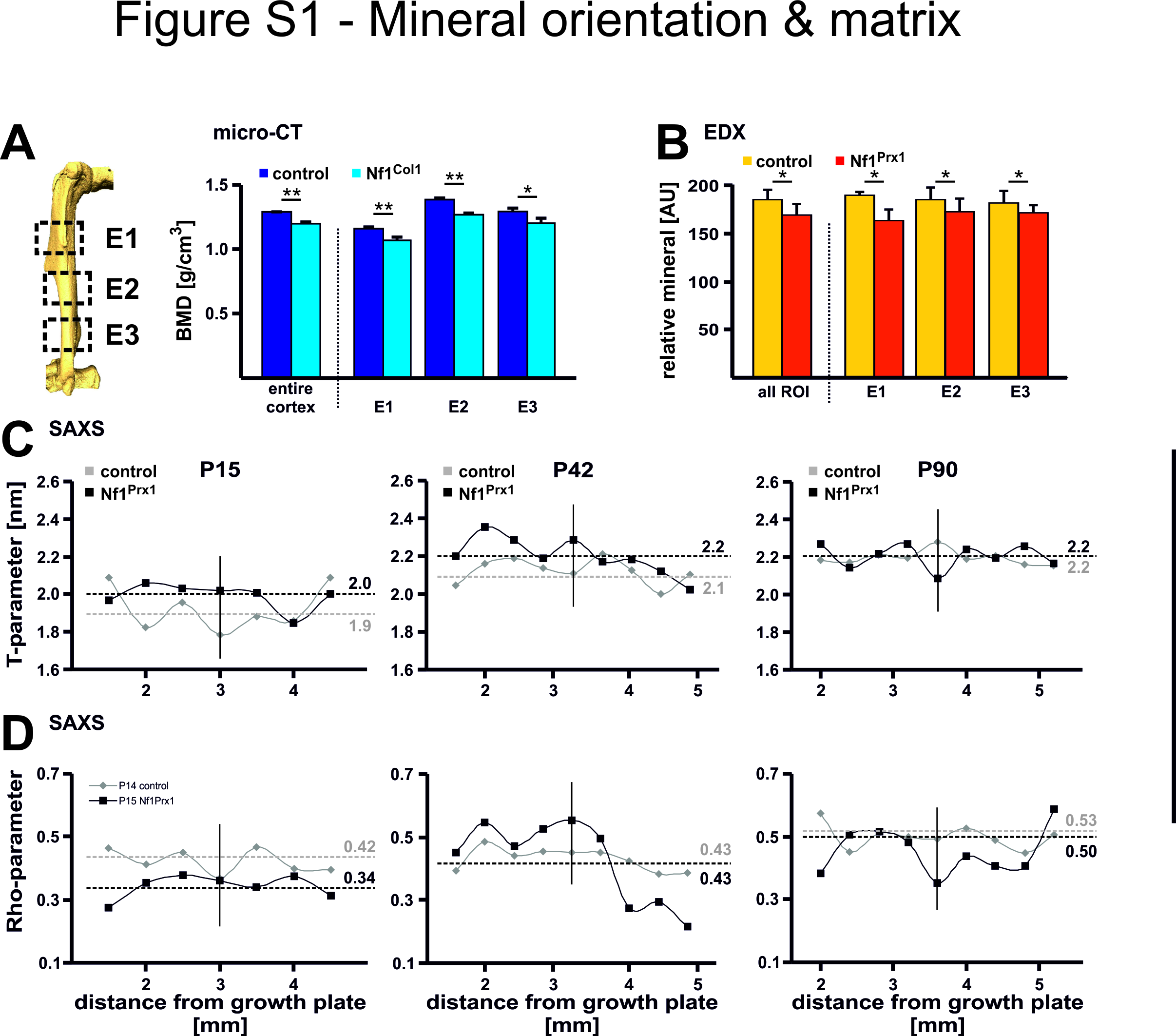

Supplement: Figure S1 — Small-angle X-ray scattering (SAXS) analysis demonstrates normal overall mineral orientation in Nf1Prx1 humerus. (A) Cortical bone mineral density (BMD) was assessed by microCT within region E1–E3 in humeri of Nf1Col1 (control n = 3, Nf1Prx1 n = 3) mice. BMD was reduced in the Nf1Col1 model in all ROIs. ROIs E1, E2 and E3 were choosen as indicated. (B) Relative mineral content was imaged by energy dispersive X-ray spectroscopy (EDX) and mean grey value intensities were measured with AxioVision (Zeiss) (controls n = 3, mutants n = 3). All cortical regions E1–E3 of Nf1Prx1 mice showed lower degree of mineralization. (C) SAXS analysis revealed normal mineral particle thickness (T-parameter) within the midshaft region of control (grey) and Nf1Prx1 (black) humeri. Analyzed samples were from different postnatal stage P15, P42 and P90. (D) The mineral particle orientation (Rho-parameter) appeared also unaffected between controls and Nf1Prx1 humerus. Position of measured points according to the distance from the growth plate is indicated at the abscissa and the vertical lines indicate centre of humerus cortex. For each developmental stage, one humerus was analyzed. Horizontal lines and adjacent numbers represent the mean value of appropriated individual measurements. Measurements at different developmental time points showed no differences between control and Nf1Prx1 humeri for the T- or Rho-parameter suggesting normal mineral particle thickness and alignment. (TIF) [file pone.0086115.s001.tif]
