# Supplementary material for: Multiscale, Converging Defects of Macro-Porosity, Microstructure and Matrix Mineralization Impact Long Bone Fragility in NF1
Source: PLoS One. 2014 Jan 21;9(1):e86115. doi: 10.1371/journal.pone.0086115 (PMC3897656; doi:10.1371/journal.pone.0086115)
Supplement: Table S1 — Cortical volume parameter in P90 humeri level E2 of control, Nf1-Prx1 and Nf1-Col1 mice measured by high resolution micro-CT. (DOC) [file pone.0086115.s002.doc]

Table S1: Cortical volume parameter in P90 humeri level E2 of control, Nf1-Prx1 and Nf1-Col1 mice measured by high resolution micro-CT.

| **value** | **unit** | **control** | **Nf1-Prx1** | **t-test** |  | **control** | **Nf1-Col1** | **t-test** |
| --- | --- | --- | --- | --- | --- | --- | --- | --- |
| **n** |  | 5 | 5 |  |  | 3 | 3 |  |
| **total cortical bone tissue volume** | µm^3^ | 700487277.8 ± 55599628.2 | 624755897.0 ± 141712038.5 | n.s. |  | 505309071.6 ± 14822222.1 | 567537682.3 ± 20841955.8 | p ≤ 0.05 |
| **sum lacunae volume**  **[range 0.05-1*10^6^ µm^3^]** | µm^3^ | 1552162.8± 437056.8 | 4826733.1 ± 583638.5 | p ≤ 0.001 |  | 1961830.8 ± 153904.5 | 2332484.9 ± 948297.5 | n.s. |
| **relative sum lacunae volume**  **[range 0.05-1*10^6^ µm^3^]** |  | 0.00221 ± 0.00059 | 0.00791 ± 0.00113 | p ≤ 0.001 |  | 0.00388 ± 0.00028 | 0.00411 ± 0.00165 | n.s. |
| **number of lacunae [with volume range 0.05-1*10^6^ µm^3^]** | n | 11.2 ± 3.8 | 23.8 ± 3.9 | p ≤ 0.001 |  | 11.7 ± 2.1 | 13.7 ± 4.7 | n.s. |
| **relative lacunae number [with volume range 0.05-1*10^6^ µm^3^]** | n/µm^3^ | 23.0*10^-9^ ± 8.0*10^-9^ | 62.0*10^-9^ ± 21.0*10^-9^ | p ≤ 0.01 |  | 28.2*10^-9^ ± 7.3*10^-9^ | 36.2*10^-9^ ± 13.4*10^-9^ | n.s. |
| **mean lacunae volume**  **[range 0.05-1*10^6^ µm^3^]** | µm^3^ | 141651.0 ± 18990.0 | 205840.0 ± 34344.4 | p ≤ 0.01 |  | 170543.9 ± 22308.0 | 170715.1 ± 34787.0 | n.s. |
|  |  |  |  |  |  |  |  |  |
| **sum lacunae volume**  **[range 10-4000 µm^3^]** | µm^3^ | 14508615.9 ± 5783704.4 | 21253082.3 ± 7523020.0 | n.s. |  | 18213007.7 ± 1691969.5 | 23273331.1 ± 404209.6 | p ≤ 0.01 |
| **relative sum lacunae volume**  **[range 10-4000 µm^3^]** |  | 0.02034 ± 0.00683 | 0.03427 ± 0.01083 | p ≤ 0.05 |  | 0.03601 ± 0.00261 | 0.04104 ± 0.00153 | p ≤ 0.05 |
| **number of lacunae [with [range 10-4000 µm^3^]** | n | 34812.8 ± 7787.4 | 34762.0 ± 6240.7 | n.s. |  | 36130.7 ± 2332.7 | 37346.3 ± 3804.3 | n.s. |
| **relative lacunae number [with [range 10-4000 µm^3^]** | n/µm^3^ | 49.3*10^-6^ ± 7.7*10^-6^ | 56.5*10^-6^ ± 7.8*10^-6^ | n.s. |  | 71.5*10^-6^ ± 3.2*10^-6^ | 65.9*10^-6^ ± 7.5*10^-6^ | n.s. |
| **mean lacunae volume**  **[range 10-4000 µm^3^]** | µm^3^ | 403.8 ± 78.9 | 599.9 ± 124.5 | p ≤ 0.05 |  | 503.5 ± 14.7 | 628.6 ± 79.3 | n.s. |
|  |  |  |  |  |  |  |  |  |

Statistical significance calculated by unpaired t-test of control vs. Nf1-Prx1/Nf1-Col1. All values are given as mean ± standard deviation.
