# Supplementary material for: Multiscale, Converging Defects of Macro-Porosity, Microstructure and Matrix Mineralization Impact Long Bone Fragility in NF1
Source: PLoS One. 2014 Jan 21;9(1):e86115. doi: 10.1371/journal.pone.0086115 (PMC3897656; doi:10.1371/journal.pone.0086115)
Supplement: Table S2 — Mechanical testing of control and Nf1-Prx1 humerus measured by tensile analysis. (DOC) [file pone.0086115.s003.doc]

Table S2: Mechanical testing of control and Nf1-Prx1 humerus measured by tensile analysis.

|  |  | **E-modulus [GPa]** | | |  | **Ultimate stress [MPa]** | | |
| --- | --- | --- | --- | --- | --- | --- | --- | --- |
| **developmental stage** | **genotype** | **n** | **mean ± stabw** | **t-test** |  | **n** | **mean ± stabw** | **t-test** |
| **P4** | control | 5 | 0.7 ± 0.9 | n.s. |  | 5 | 14.9 ± 13.1 | n.s. |
| **P4** | Nf1-Prx1 | 5 | 0.3 ± 0.2 |  |  | 5 | 15.7 ± 6.0 |  |
| **P18** | control | 3 | 2.8 ± 1.0 | p ≤ 0.05 |  | 3 | 31.1 ± 20.2 | n.s. |
| **P18** | Nf1-Prx1 | 5 | 0.9 ± 0.6 |  |  | 5 | 28.2 ± 10.2 |  |
| **P60** | control | 2 | 6.4 ± 0.3 | n.s. |  | 2 | 74.0 ± 31.2 | n.s. |
| **P60** | Nf1-Prx1 | 2 | 3.3 ± 2.2 |  |  | 2 | 51.9 ± 6.8 |  |
| **P90** | control | 15 | 27.5 ± 9.9 | p ≤ 0.001 |  | 15 | 103.6 ± 35.8 | p ≤ 0.005 |
| **P90** | Nf1-Prx1 | 14 | 15.0 ± 6.9 |  |  | 14 | 67.8 ± 27.5 |  |
| **P120** | control | 3 | 27.8 ± 14.3 | n.s. |  | 3 | 103.4 ± 34.6 | n.s. |
| **P120** | Nf1-Prx1 | 3 | 11.0 ± 3.4 |  |  | 3 | 47.2 ± 11.5 |  |
|  |  |  |  |  |  |  |  |  |

Statistical significance calculated by t-test of control vs. Nf1-Prx1 of each age. All values are given as mean ± standard deviation.

Sections for tensile analysis were generated by laser dissection.
