# Supplementary material for: Multiscale, Converging Defects of Macro-Porosity, Microstructure and Matrix Mineralization Impact Long Bone Fragility in NF1
Source: PLoS One. 2014 Jan 21;9(1):e86115. doi: 10.1371/journal.pone.0086115 (PMC3897656; doi:10.1371/journal.pone.0086115)
Supplement: Table S3 — Acoustic impedance measurement of P90 humeri of control, Nf1-Prx1 and Nf1-Col1 mice measured by ultrasound microscopy. (DOC) [file pone.0086115.s004.doc]

Table S3: Acoustic impedance measurement of P90 humeri of control, Nf1-Prx1 and Nf1-Col1 mice measured by ultrasound microscopy.

| **value** | **unit** | **control** | **Nf1-Prx1** | **ANOVA** |  | **control** | **Nf1-Col1** | **ANOVA** |
| --- | --- | --- | --- | --- | --- | --- | --- | --- |
| **n** |  | 4 | 4 |  |  | 5 | 4 |  |
| **E1+E2+E3** | MRayl | 6.83 ± 0.59 | 5.96 ± 0.70 | p ≤ 0.001 (F=21.8) |  | 5.92 ± 0.69 | 5.90 ± 0.73 | n.s. |
| **Anterior** | MRayl | 6.60 ± 0.64 | 5.89 ± 0.71 | p ≤ 0.05 (F=6.7) |  | 5.76 ± 0.65 | 5.83 ± 0.83 | n.s. |
| **Posterior** | MRayl | 7.06 ± 0.46 | 6.03 ± 0.70 | p ≤ 0.001 (F=17.9) |  | 6.09 ± 0.72 | 5.98 ± 0.66 | n.s. |
| **E1** | MRayl | 6.26 ± 0.48 | 5.89 ± 0.76 | n.s. |  | 5.36 ± 0.55 | 5.20 ± 0.45 | n.s. |
| **E2** | MRayl | 6.98 ± 0.51 | 5.97 ± 0.71 | p ≤ 0.01 (F=10.6) |  | 6.26 ± 0.56 | 5.89 ± 0.51 | n.s. |
| **E3** | MRayl | 7.25 ± 0.26 | 6.18 ± 0.57 | p ≤ 0.001 (F=21.9) |  | 6.15 ± 0.62 | 6.62 ± 0.40 | n.s. |
| **E1-A** | MRayl | 5.92 ± 0.33 | 5.70 ± 0.82 | n.s. |  | 5.19 ± 0.50 | 5.09 ± 0.51 | n.s. |
| **E2-A** | MRayl | 6.69 ± 0.45 | 5.89 ± 0.83 | n.s. |  | 6.06 ± 0.46 | 5.80 ± 0.67 | n.s. |
| **E3-A** | MRayl | 7.19 ± 0.30 | 6.06 ± 0.63 | p ≤ 0.05 (F=10.5) |  | 6.02 ± 0.65 | 6.60 ± 0.53 | n.s. |
| **E1-P** | MRayl | 6.59 ± 0.34 | 6.08 ± 0.76 | n.s. |  | 5.53 ± 0.61 | 5.31 ± 0.42 | n.s. |
| **E2-P** | MRayl | 7.26 ± 0.44 | 6.04 ± 0.70 | p ≤ 0.05 (F=8.8) |  | 6.45 ± 0.63 | 5.99 ± 0.36 | n.s. |
| **E3-P** | MRayl | 7.32 ± 0.23 | 5.97 ± 0.86 | p ≤ 0.05 (F=9.0) |  | 6.28 ± 0.65 | 6.64 ± 0.31 | n.s. |

Statistical significance calculated by ANOVA analysis of Nf1-Prx1 and Nf1-Col1 mice vs. appropriated controls. All values are given as mean ± standard deviation.

Multiple factor ANOVA showed in Nf1-Prx1 (F=24.2) but not Nf1-Col1 humeri significantly lower acoustic impedance compared to appropriated controls.

Please note lower impedance values in region E1 compared to E2 and E3 in both mouse models.
